# Ultrasonographic diagnosis of bilateral partial rupture of the infraspinatus muscle in a racing greyhound

**DOI:** 10.29374/2527-2179.bjvm003120

**Published:** 2021-04-23

**Authors:** Maria Ligia de Arruda Mestieri, Bruna Gonzalez dos Santos, Mayara Nóbrega Gomes da Silva, João Paulo da Exaltação Pascon, Leandro Ziemer Carneiro

**Affiliations:** 1 Veterinarian, Programa de Pós-Graduação em Ciência Animal, Universidade Federal do Pampa (UNIPAMPA), Uruguaiana, RS, Brazil.; 2 Veterinarian, DSc., autonomous, Uruguaiana, RS, Brazil.

**Keywords:** ultrasonography, orthopedics, dog, muscle injury, sports, ultrassonografia, ortopedia, cão, lesão muscular, esportes

## Abstract

Athletic dogs are more susceptible to musculoskeletal disorders, especially in the muscles or tendons surrounding the shoulder joint. In this report, we describe a case of partial bilateral rupture of the infraspinatus muscle, an unusual injury. Clinical signs included lameness of the left thoracic limb after training. On physical examination, there was discomfort on bilateral palpation in the infraspinatus fossa region. The main clinical suspicion was partial rupture or myositis of the infraspinatus muscle (IF). Ultrasound examination of the region revealed the presence of hypoechoic areas intermingling the muscle fibers in a deeper and distal region of the infraspinatus muscle, compatible with edema or bilateral intramuscular hematoma (left +++, right ++) and integrity of the fascia. The findings were characterized as a partial rupture of the musculature. A detailed physical examination associated with ultrasound examination is essential for early diagnosis and therapeutic management. Although no other reports of partial rupture of the IF have been found, partial or complete rupture of muscle fibers in sport dogs is frequent. Early diagnosis prevents the development of contractures due to the institution of therapy before the fibrous process is established and improves prognosis.

## Resumo

Os cães atléticos são mais suscetíveis a sofrer de distúrbios musculoesqueléticos, especialmente nos músculos ou tendões ao redor da articulação do ombro. Neste relato, é descrito um caso de ruptura parcial bilateral do músculo infraespinhal, uma lesão incomum. Os sinais clínicos incluíram claudicação no membro torácico esquerdo após o treinamento de corrida. Ao exame físico, havia desconforto à palpação bilateral em região da fossa infraespinhal. A principal suspeita clínica foi ruptura parcial ou miosite do músculo infraespinhal (IF). O exame ultrassonográfico da região revelou a presença de áreas hipoecóicas mesclando as fibras musculares em região mais profunda e distal do músculo infraespinhal, compatível com edema ou hematoma intramuscular bilateral (esquerdo +++, direito ++) e integridade da fáscia. Os achados foram caracterizados como ruptura parcial da musculatura. O exame físico detalhado associado ao exame de ultrassom foi essencial para o diagnóstico precoce e o manejo terapêutico. Embora nenhum outro relato de ruptura parcial do IF tenha sido encontrado, rupturas parciais ou totais das fibras musculares em cães esportivos são frequentes. O diagnóstico precoce evita o desenvolvimento de contraturas devido à instituição da terapia antes do estabelecimento do processo fibroso e melhora o prognóstico.

## Introduction

Over the past decade, breeding dogs for athletic purposes such as racing, agility, and flyball has become more popular and common worldwide (Baltzer, 2012). Athletic dogs are more susceptible to musculoskeletal disorders, especially muscle or tendon injuries involving the shoulder joint (Baltzer, 2012). According to Aydemir et al. (2010), the diagnosis of muscle and tendon injuries, such as ruptures, inflammation, and contractures, can be performed using ultrasonography (US) and/or magnetic resonance imaging (MRI) (Aydemir et al., 2010). However, the diagnosis of shoulder injuries that result in lameness is challenging (Murphy et al., 2008).

The present study aimed to describe the ultrasonographic findings of bilateral partial rupture of the infraspinatus muscle in a racing Greyhound.

## Case description

A 3-year-old male greyhound dog weighing 31 kg presented to the Veterinary Hospital with lameness after race and training. The animal was trained daily, including a warm-up of 7 minutes followed by high-speed 250-m races. In addition, without any medical recommendations, the dog received three doses of androgenic steroids (Estrombol®). The last application occurred 5 months prior to the reported consultation. Three months before consultation, the dog’s owner noticed occasional bilateral forelimb lameness immediately after racing and training. The lameness was progressively worse and more frequent on the left forelimb, with a performance decrease in the last two weeks.

In general and on orthopedic physical examinations, the presence of lameness was not observed. Pain and discomfort were bilaterally evidenced only during palpation of the infraspinatus fossa region. The differential diagnosis was partial rupture or myositis of the infraspinatus muscle (IF).

**Figure 1:**
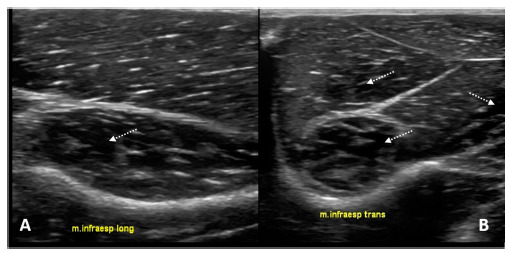
Ultrasonographic transversal views, using a 10-MHz transducer, of the infraspinatus muscle (IF) of a greyhound racing dog. (A) In the right IF, hypoechoic areas interspersed into the muscle fibers are detectable (arrow); (B) In the left IF, those findings are more evident and severe (arrows).

Triage radiography (mediolateral projection) and ultrasonographic examination were performed. No notable radiographic changes were observed. Ultrasonographic examination (Figure 1) revealed muscle fiber discontinuity, interspersed with indefinite margins of hypoechoic areas at the deepest parts of the IF (left +++, right ++). The infraspinatus tendons, biceps brachii, supraspinatus muscles and tendons, and brachial plexus were identified and showed no alterations. Thus, the animal was sent to the clinical department for proper treatment.

After 30 days of clinical treatment including rest, carprofen (2.2 mg/kg BID per 21 days), and therapeutic ultrasound in a reference physiotherapy clinic, the dog returned for inspection. Physical examination revealed no signs of lameness, restriction of movement, or pain on palpation. The dog progressively returned to training activities.

## Discussion

A study involving 767 racing greyhounds corroborated the findings by demonstrating that racing dogs suffer mostly from forelimb injuries (75%) (Marcellin-Little et al., 2005). It was also asserted that orthopedic injuries in athletic dogs are commonly observed due to an increased activity applied to bones, joints, muscles, tendons, and ligaments (Marcellin-Little et al., 2005). IF is responsible for stabilizing the shoulder joint, and the long head of the triceps brachii muscle (LHTH) is required for stretching and shortening the cycles of exercise (Cullen et al., 2017).

Thus, studies have shown that LHTH and IF are mostly required to meet the racing activity demand and, consequently, are the most vulnerable injured muscles during this activity (Cullen et al., 2017). Although IF is reported to be rarely affected by lesions, it seems to be very important for racing dogs, which corroborates the findings of Cullen et al. (2017). However, since the patient did not present with pain or alteration on physical examination of the triceps brachii muscle area, ultrasonography was not performed in this region.

In addition to the racing activity, this patient showed one more predisposing factor for muscle injury. The use of androgenic steroids is responsible for dramatically increasing muscular mass in physically active individuals (Casavant et al., 2007). Increased muscle mass can lead to a higher susceptibility of muscles to rupture. Similar studies in dogs were not conducted in the present review. Nevertheless, the use of androgenic steroids associated with intense exercise could contribute to the occurrence of bilateral IF muscle injury in this case.

Although IF injuries usually do not result in clinical signs and pain on palpation, affected dogs can present progressive lameness (Baltzer, 2012), leading the athlete animal to be indefinitely absent from its activities. In the present report, partial rupture of the IF resulted in episodes of lameness after training. No other cases of partial rupture of IF have been reported. A case of simultaneous bilateral contracture of the infraspinatus muscle was previously reported (Franch et al., 2009), which is also an uncommon condition in dogs (Taylor & Tangner, 2007).

It is believed that contractures of the infraspinatus muscle are consequences of degeneration and fibrosis (Franch et al., 2009) after muscular tears and hemorrhage. These lesions may be caused by forceful stretching during contraction, not by a single event, but mostly associated with continuous small trauma, perhaps caused by inadequate training or warm-up (Devor & Sørby, 2006; Pettit et al., 1978). Therefore, early diagnosis and therapy of less obvious injuries, such as partial tears, are important to prevent contractures.

IF contracture is irreversible and cannot be treated conservatively, leading to the need for infraspinatus tenotomy, with serious consequences for an athletic dog. In addition, for fibrotic contracture diagnosis, ultrasonographic findings are crucial: disorganized hyperechogenic areas in both infraspinatus muscles and loss of muscular fiber continuity compared to the more normal pattern of the supraspinatus muscle are observed (Franch et al., 2009).

It is important to distinguish contractures from partial ruptures of the IF. In muscle partial rupture, discontinuity of the fibers and anechoic areas caused by the presence of intramuscular hematoma is seen on ultrasound. However, it can progress to fibrotic contracture (Ferreira & Mistieri, 2019). In the present report, ultrasonography showed muscle fiber discontinuity, interspersed with indefinite margins of hypoechoic areas at the deepest parts of the IF. These findings were compatible with bilateral intramuscular edema or hematoma and muscular fiber disruption, consistent with partial rupture of the IF. Areas with increased echogenicity were not observed, indicating fibrosis formation.

Ultrasonography is reported to be as efficient as MRI for diagnosing muscle rupture (Aydemir et al., 2010). However, it would be possible to obtain more valuable information about this type of lesion extension using MRI (Aydemir et al., 2010). Although MRI was not available, ultrasound examination was highly useful for diagnosing this muscle lesion and determining its extension. In addition, the diagnosis of this case was made at an early stage, and it is unknown if it would have evolved to muscle contracture if no proper treatment was prescribed.

## Conclusion

It is believed that the practice of intense physical activity and the concurrent use of androgenic steroids may have predisposed the dog to develop IF injury. Detailed physical and ultrasonographic examinations were essential for the early-stage diagnosis of partial rupture of IF in this case, allowing for proper clinical treatment and rehabilitation.
